# The role of aspirin in carcinogenesis

**DOI:** 10.1038/sj.bjc.6600670

**Published:** 2002-11-12

**Authors:** G J Caine, S T Kehoe, G Y H Lip

**Affiliations:** University Department of Medicine, City Hospital, Birmingham B18 7QH, UK; Department of Gynaecological Oncology, Birmingham Women's Hospital, Birmingham B15 2TG, UK

## Abstract

*British Journal of Cancer* (2002) **87**, 1337–1338. doi:10.1038/sj.bjc.6600670 www.bjcancer.com

© 2002 Cancer Research UK

## 

**Sir**

We read with interest the excellent paper by Akhmedkhanov *et al* (2002) regarding aspirin use and the incidence of lung cancer. We would like to offer another possible anti-cancer property of aspirin, namely, the inhibition of phenolsulphotransferase (PST) activity.

PSTs are found throughout the body, but the bowel, liver and platelets are known to contain particularly high activities of this enzyme. PSTs are cytosolic and exist in two forms: (i) P-PST, which selectively sulphates micromolar concentrations of phenols; and (ii) M-PST, which is similarly selective for aromatic amines.

The main function of this sulphation is to scavenge low concentrations of endogenous and exogenous toxins from the body, but the lability of the phenolic sulphate-ester bond means it is liable to cause the formation of electrophilic free radicals. These react chemically with DNA which may cause mutations leading to neoplasia (Coughtrie, 1996).

Food cooking can result in a wide variety of mutagenic compounds, including polyaromatic hydrocarbons and heterocyclic amines, especially if the food becomes charred when grilled or barbequed. Certainly, several polyaromatic hydrocarbons have been shown to be activated by hydroxylation to phenols followed by sulphation via P-PST to the final mutagenic form (Grover, 1986). P-PST has also been found to be responsible for the activation of heterocyclic amines by N-sulphation, for example, the bladder carcinogen 2-napthylamine (Hernandez *et al*, 1991) and a wide variety of carcinogenic N-hydroxy arylamines (Chou *et al*, 1995).

Thus, inhibition of P-PST would block one route of activation for both main groups of carcinogen found in food. Indeed, Rao and Duffel (1991) have shown that salicylic acid, the initial breakdown product of aspirin, is a potent inhibitor of aryl sulphotransferase IV (AST IV), at least in the rat – and AST IV is the rat equivalent of human PST enzymes. Other studies (Boberg *et al*, 1983; Tsutumi *et al*, 1995) have also shown that sulphotransferase inhibitors dramatically reduces the potency of sulphation activated carcinogens in both mice and hamsters.

We would therefore suggest that the action of salicylic acid on P-PST, by preventing the excessive activation of carcinogens, may represent another possible pathway by which aspirin may reduce cancer risk.
